# Transnasal Sphenopalatine Ganglion Block for the Preventive Treatment of Chronic Daily Headache in Adolescents

**DOI:** 10.3390/children8070606

**Published:** 2021-07-18

**Authors:** Megan Kouri, Marta Somaini, Victor Hugo González Cárdenas, Kacper Niburski, Marie Vigouroux, Pablo Ingelmo

**Affiliations:** 1 Faculty of Medicine, McGill University, Montreal, QC H3G 2M1, Canada; megan.kouri@mail.mcgill.ca (M.K.); kacper.niburski@mail.mcgill.ca (K.N.); 2 Department of Anaesthesia, Grande Ospedale Metropolitano Niguarda, 201262 Milano, Italy; ma.somaini@gmail.com; 3 Faculty of Medicine, Fundación Universitaria de Ciencias de la Salud (FUCS), 111221 Bogotá, Colombia; vhgonzalez@fucsalud.edu.co; 4 Department of Anesthesia, Los Cobos Medical Center, 110121 Bogotá, Colombia; 5 Department of Anesthesia, Pain and Palliative Care, Hospital Universitario de la Samaritana, 110411 Bogotá, Colombia; 6 Faculty of Dentistry, McGill University, Montreal, QC H3A 1G1, Canada; marie.vigouroux@mail.mcgill.ca; 7 Edwards Family Interdisciplinary Center for Complex Pain, Montreal Children’s Hospital, McGill University Health Center, Montreal, QC H4A 3J1, Canada; 8 The Alan Edwards Centre Research on Pain, McGill University, Montreal, QC H3A 0G1, Canada

**Keywords:** chronic headache, migraine, sphenopalatine ganglion, Tx360^®^, adolescents, chronic pain

## Abstract

Chronic headaches are a major source of morbidity in the pediatric population, affecting physical function, school attendance, social capacity, mood, and sleep. In adults, repetitive sphenopalatine ganglion (SPG) blockade has been studied as a preventive treatment for chronic migraines. This case series aims to evaluate the SPG block for the preventive treatment of chronic daily headache (CDH) in adolescents. We prospectively evaluated 17 adolescents (14 females, 14 ± 1 year) with CDH not responding to cognitive behavioral therapy (CBT), physiotherapy, and standard medications. Each patient received 10 SPG blocks (two blocks/week) using the Tx360^®^ device. At the end of treatment, 10 patients (59%) reported a Patient’s Global Impression of Change (PGIC) score ≥ 67%, and 3 months after the end of treatment, nine patients (53%) sustained a PGIC ≥ 67%. There was also a statistically significant reduction in the depression subscale of the Revised Children’s Anxiety and Depression Scale (RCADS) at the end of treatment and 3 months post-treatment compared with baseline. The procedure was well tolerated with no adverse effects. In our study, the use of repeat SPG blockade was associated with sustained benefits on the PGIC and the depression subscale of the RCADS when used as preventive headache treatment in adolescents with refractory CDH.

## 1. Introduction

Headaches are a common neurological disorder in children, affecting as many as 88% of the pediatric population [1]. Up to 4% of children and adolescents suffer from headaches included in the umbrella term “chronic daily headache” (CDH), defined as having at least 15 headache days per month [2]. Chronic headaches reduce quality of life by affecting school attendance, social capacity, physical function, mood, and sleep [1,3,4]. Preventive medications are indicated for patients with significant headache-related disability, but the majority of randomized controlled trials studying preventive medications fail to demonstrate superiority to placebo [1,5]. In addition, most preventive medications commonly prescribed have undesirable adverse effects [6].

The sphenopalatine ganglion (SPG), also known as the pterygopalatine ganglion, is a complex parasympathetic structure that has been successfully targeted to treat various headache disorders and facial pain syndromes in adults [7]. Located in the pterygopalatine fossa, the SPG is easily accessible through the nose below the middle turbinate [8]. SPG blocks have been performed for decades, using techniques of varying invasiveness and accuracy [9]. In recent years, a non-invasive medication delivery device called the Tx360^®^ (Tian Medical Inc., Lombard, IL, USA) was developed specifically to access the SPG through the nose. The device allows for more patient comfort as compared to a cotton-tip swab, which has been used historically for transnasal SPG access [9]. In adults, repeat SPG blockade with bupivacaine delivered with the Tx360^®^ device reduced number of headache days and improved quality of life in chronic migraine patients [8]. 

While there is an abundance of literature regarding SPG blockade in adults, research in the pediatric population is lacking. In 2017, Kaye et al. described the use of SPG blocks for acute treatment of chronic migraine in youth aged 7 to 18 and found a significant decrease in headache pain 10 min after the procedure [10]. These results suggest this technique as a potential abortive treatment for acute headache relief in children but do not provide information on its use for headache prevention. Moreover, there are currently no studies describing repeat SPG blockade for chronic headache relief in the pediatric population. In this prospective case series, we aim to describe the effectiveness of repetitive SPG blockade using the Tx360^®^ as a preventive treatment for CDH in adolescents.

## 2. Materials and Methods

Ethical Approval—The IRB of the McGill University Health Center approved this prospective study (2019-4887). Informed consent was obtained from the patient and a parent prior to each procedure. 

Study Population—The study population consisted of patients under 18 years old enrolled in the interdisciplinary program of the Chronic Pain Service at the Montreal Children’s Hospital. Patients were recruited to the study if they had a diagnosis of CDH, defined as having at least 15 headache days per month for at least 3 months, and did not respond to the standard multidimensional treatment program, which consists of physiotherapy, cognitive behavioral therapy (CBT), and pharmacological treatments including abortive and preventive medications. Patients were excluded from the study if they had a previously known nasal deformity or pathology, allergy to local anesthetics, bleeding disorder, previous intolerance to transnasal access, or were unavailable to attend all treatment visits. 

Interventions—At each treatment visit, a medical doctor administered 0.3 mL of 0.5% bupivacaine through each nostril using the Tx360^®^ device with the patient in the upright seated position. The device is a single-use sterile system consisting of a syringe and catheter with a soft tip [11]. The catheter tip is directed posteriorly to the inferior nasal turbinate, where it sprays bupivacaine superiorly, laterally, and anteriorly to bathe the SPG [12]. Each procedure took less than 30 s, and patients were discharged within minutes to resume normal activities. Each patient received two SPG blocks per week over 5 weeks for a total of 10 blocks. This frequency was chosen based on a previous study on adults with chronic migraines [8]. Throughout the treatment period, patients continued all aspects of the multidisciplinary program specific to each individual. As part of our program, all patients are enrolled in CBT and physiotherapy with a specialized psychologist and physical therapist, respectively. Patients continued with their CBT and physiotherapy throughout the treatment period. Patients were instructed to continue taking their medications as usual, and no restrictions were made with regard to medication adjustments or changes throughout the treatment period. 

Outcome Measures—The primary endpoint of this study was the proportion of patients scoring 67% or higher on the Patient’s Global Impression of Change (PGIC) scale (Table 1) [13]. The PGIC is a self-reported measure that reflects overall improvement as perceived by the patient [14]. At the end of treatment and 3 months after treatment, patients were asked: “Since beginning treatment, how would you describe the change (if any) in activity limitations, symptoms, emotions and overall quality of life related to your painful condition?” [13]. Patients chose one from the seven provided responses to this question, where each corresponds to a percentage of perceived improvement. We considered a meaningful improvement to be a PGIC of 67% or greater. Responses 5, 6, and 7 represent scores ≥ 67%.

The secondary endpoints measured were anxiety and depression assessed with the Revised Children’s Anxiety and Depression Scale (RCADS), physical function assessed with the Functional Disability Inventory (FDI), and sleep quality assessed with the Pittsburgh Sleep Quality Index (PSQI). The RCADS is a valid self-report Likert scale questionnaire that measures the frequency of anxiety and depression symptoms in pediatric patients [15]. Its five subscales are separation anxiety disorder, social phobia, generalized anxiety disorder, panic disorder, obsessive–compulsive disorder, and depression. A higher subscale score suggests more frequent behavior related to that subscale. The FDI is commonly used as a measure of physical function and disability in youth with chronic pain [16]. This questionnaire requires the patient to rank different activities, such as walking up the stairs and completing homework, on a Likert scale from being impossible (4) to being no trouble (0) to complete. A higher score is indicative of a higher degree of disability. The PSQI is a widely used self-report questionnaire used to assess general sleep quality [17]. One portion asks quantitative values relating to sleep, such as time spent in bed and time spent asleep. Then, a Likert scale asks the frequency of various sleep disturbances (e.g., bad dreams, needing to get up to use the bathroom), use of sleep medications, and daytime dysfunction. A calculation using both components provides a score such that a higher score indicates poorer sleep quality [18]. Outcome measures were evaluated before the first SPG block (baseline), after the tenth block (end of treatment) and 3 months after completion of treatment. 

*Statistical Analysis*—Results were expressed as frequencies (n) and proportions (%) for qualitative data (gender, diagnoses, PGIC, side effects). Median (and interquartile range) and/or mean (and standard deviation) were used for quantitative variables (age, RCADS score, FDI score, PSQI score) according to the normality distribution test. Normality was tested using the Shapiro–Wilk test. Fisher’s exact test for dichotomous variables and a two-sided *t*–Student test or Mann–Whitney U test for continuous quantitative variables (according to its normality test). A *p*-value of <0.05 was considered significant. All analyses were performed with IBM SPSS^®^ statistical software version 25 March 2017. 

## 3. Results

### 3.1. Patient Demographics

A total of 17 patients completed the SPG block series, including 14 (82%) females and three males. The mean age at the start of treatment was 14 (11–17) years. Fifteen (88%) patients had a diagnosis of chronic primary headache or migraine, and two patients had chronic headache secondary to concussion. All patients completed the blocks series. Throughout the treatment period, three patients (18%) had a change of medication, consisting of two dosage adjustments to preexisting medications and one new medication added. 

### 3.2. Primary Endpoint

**Figure 1:**
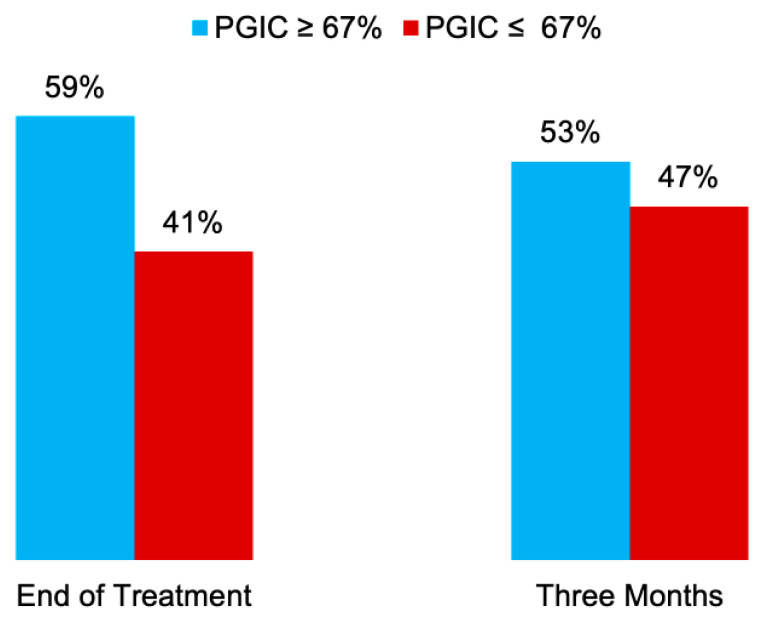
Proportion of patients reporting a PGIC ≥ 67% at the end of treatment and 3 months after the end of treatment. PGIC, Patient’s Global Impression of Change.

Ten of seventeen patients (59%) reported a PGIC of 67% or higher at the end of treatment, and nine patients (53%) reported a PGIC of 67% or higher 3 months after the end of treatment (Table 2, Figure 1). 

### 3.3. Secondary Endpoints

In five out of six subscales of the RCADS score, there was no improvement at either time point after treatment. There was a significant decrease in the depression subscale both immediately after (*p* = 0.057) and 3 months after treatment (*p* = 0.026). There were no changes in the FDI or PSQI scores (Table 3).

### 3.4. Side Effects

All patients experienced a mildly unpleasant taste in the mouth due to the bitterness of bupivacaine, which was controlled with candy or gum immediately after the procedure. The procedure was otherwise well-tolerated.

## 4. Discussion

This prospective case series describes the effectiveness of repetitive transnasal SPG blockade using the Tx360^®^ with 0.5% bupivacaine as a preventive treatment for CDH in adolescents. Just over half of the patients reported a meaningful benefit (PGIC ≥ 67%) up to 3 months after completing treatment. There was also a clinically significant decrease in depression symptoms up to 3 months after treatment.

The presence of symptom improvement several months after completion of the SPG block series was also observed in adults. A double-blind, placebo-controlled study was conducted to determine if repetitive SPG blockade using the Tx360^®^ has long-term benefits in adults with chronic migraines [8]. Thirty-eight subjects were randomized to receive 0.3 mL of either 0.5% bupivacaine or saline in each nostril twice a week for 6 weeks. The bupivacaine group had a decrease in average pain from pre-treatment to the end of treatment, 1 month after treatment, and 6 months after treatment. Headache impact scores and quality of life measures were also improved up to 6 months after treatment. Our study showed a significant decrease in depression symptoms, and while the adult study did not measure specific psychologic symptoms, it did find an improvement in mood interference ratings at 1 and 6 months post-treatment. 

Our choice of local anesthetic was chosen based on the previously mentioned study in adults with chronic migraine [8]. However, various agents are used for SPG blocks, including local anesthetics, steroids, phenol, or a combination [19,20]. There are few head-to-head studies comparing the efficacy of different medications, and the existing findings fail to demonstrate a superior agent. A retrospective study evaluating 386 SPG blocks for headache found no difference between bupivacaine and lidocaine for pain reduction [21]. A randomized controlled study of 90 patients compared SPG block with lidocaine 2%, lidocaine 5%, and bupivacaine 0.5% for treating post-dural puncture headache and again found no difference between the groups [22]. 

For optimal pediatric pain management, research supports a multidisciplinary care model, and a comprehensive approach may include patient education, behavioral therapies, and medical intervention [2,23,24]. While a multidisciplinary approach is ideal, access to complex pain management programs is limited [25]. 

CBT with a focus on pain coping skills has been shown to improve headache frequency and intensity in children and adolescents with CDH [2,26]. However, CBT is not always feasible due to the required time commitment and barriers to access, such as geographical location and cost [27].

The American Academy of Neurology published a systematic review in which the authors found that no preventive treatment alone had high confidence evidence in improving outcomes in pediatric chronic migraine [5]. The recommendations made include counseling on lifestyle and behavioral factors that affect headache frequency and management of comorbid disorders. In addition to insufficient evidence for efficacy, medications commonly prescribed have many potential side effects [6,23,28]. In children with CDH, poor compliance with taking preventive medications may be a factor in treatment response [2]. The poor risk-to-benefit ratio and importance of compliance of preventive medications used in pediatric CDH are discouraging and may increase the appeal of interventional treatment. Onabotulinumtoxin A injections are sometimes used off-label as an interventional treatment for pediatric migraine prophylaxis [29]. However, there is insufficient evidence of efficacy, and the number of needles needed per session can be deterring to young patients [5,29].

Though this case series does not establish efficacy, repeat SPG blockade using bupivacaine and the Tx360^®^ has potential as a preventive treatment for adolescents with refractory CDH. A small majority of our patients had significant subjective improvement, which was sustained 3 months after treatment, as well as reduced symptoms of depression. This intervention was well-tolerated with minimal discomfort in our case series. It may be an attractive alternative to existing interventional procedures due to its lack of needles and simplicity of use. 

This study has several limitations, the main ones being a small sample size (N = 17) and lack of a control group. Pediatric migraine treatment trials have a considerable placebo effect, with one systematic review finding 30–61% of children receiving placebo to have a 50% or greater reduction in headache frequency [5]. It is therefore likely that the placebo effect contributed to some of the improvement seen in our study. In addition, the care of each patient is multifaceted, including CBT, physiotherapy, and medications. The extent to which these treatments contributed to patient improvement is unknown, and it is possible there was a synergistic effect. 

Our primary endpoint was the PGIC which is a scale that quantifies clinical improvement in the patient’s condition and overall subjective quality of life. Many studies, however, use the PedMIDAS score to assess disability caused by pediatric and adolescent headaches, which was derived from the widely used adult MIDAS score [30]. Another standard outcome measure used in headache research is the number of headache days, which we did not evaluate. Our clinic focuses on the quality of life and function of the patient, which reflects our chosen outcome measures. We do not encourage patients nor their parents to keep a headache diary so as to avoid fixation on the child’s pain; therefore, we did not measure the number of headache days in our study. 

This pilot study assessed outcomes 3 months after treatment but does not provide information on the long term. It is unknown if the blocks would eventually need to be repeated, and if so, at what frequency. 

Lastly, the small population we studied consisted of particularly challenging cases of CDH as these patients had previously failed other treatments. Due to the many limitations of this study, firm conclusions regarding this technique cannot be drawn. A randomized, double-blind, placebo-controlled study of repeat SPG blockade using the Tx360^®^ in adolescents with CDH is needed. 

## 5. Conclusions

In this study, half the adolescents with CDH not responding to a multidisciplinary pain treatment program perceived meaningful benefits, which lasted up to 3 months after repetitive SPG blockade with the Tx360^®^. The procedure was well-tolerated with minimal discomfort and no adverse effects. The results of this study should be extrapolated cautiously given the small sample size, lack of placebo control, and use of concomitant treatments during the studied treatment period, such as CBT and medications. Further research is warranted. 

## Figures and Tables

**Table 1:** Patient’s Global Impression of Change Scale (PGIC) [13].

PGIC		
1	No change (or condition has got worse)	0%
2	Almost the same, hardly any change at all	17%
3	A little better, but no noticeable change	34%
4	Somewhat better, but the change has not made any real difference	50%
5	Moderately better, and a slight but noticeable change	67%
6	Better, and a definite improvement that has made a real and worthwhile difference	84%
7	A great deal better, and a considerable improvement that has made all the difference	100%

**Table 2:** PGIC scores at the end of treatment and 3 months after the end of treatment.

PGIC	End of Treatment (N = 17)	3 Months (N = 17)
0%	2	1
17%	3	1
34%	0	3
50%	2	3
67%	5	3
84%	3	4
100%	2	2

PGIC, Patient’s Global Impression of Change.

**Table 3:** Median and range RCADS, FDI, and PSQI at baseline, after treatment, and 3 months after treatment.

	Baseline	End of Treatment	3 Months
RCADS			
Separation Anxiety	50 (42.5–65)	50 (43.5–63)	50 (46–63)
General Anxiety	41 (34–50)	41 (31.5–44.5)	39 (33–46)
Panic	47 (39–61)	47 (41–57.5)	47 (40–55.5)
Social Phobia	31 (27–39)	28 (26–35)	26 (24–34)
Obsessions/Compulsions	49 (43.5–53.5)	46 (37–54)	49 (40.5–52)
Depression	58 (40–60)	42 (37–52.5)	45 (34.5–47.5)
FDI	17 (7.5–29.5)	12 (3.5–20.5)	17 (5–24)
PSQI	8 (6–9.5)	6.5 (4–10.25)	9 (3–12)

RCADS, Revised Children’s Anxiety and Depression Scale; FDI, Functional Disability Inventory; PSQI, Pittsburgh Sleep Quality Index.

## References

[1] Langdon R., DiSabella M.T. (2017). Pediatric Headache: An Overview. Curr. Probl. Pediatr. Adolesc. Health Care.

[2] Connelly M., Sekhon S. (2019). Current perspectives on the development and treatment of chronic daily headache in children and adolescents. Pain Manag..

[3] Mack K.J. (2006). An approach to children with chronic daily headache. Dev. Med. Child Neurol..

[4] Koller L.S., Diesner S.C., Voitl P. (2019). Quality of life in children and adolescents with migraine: An Austrian monocentric, cross-sectional questionnaire study. BMC Pediatr..

[5] Oskoui M., Pringsheim T., Billinghurst L., Potrebic S., Gersz E.M., Gloss D., Holler-Managan Y., Leininger E., Licking N., Mack K. (2019). Practice guideline update summary: Pharmacologic treatment for pediatric migraine prevention: Report of the Guideline Development, Dissemination, and Implementation Subcommittee of the American Academy of Neurology and the American Headache Society. Neurology.

[6] Teleanu R.I., Vladacenco O., Teleanu D.M., Epure D.A. (2016). Treatment of Pediatric Migraine: A Review. J. Clin. Med..

[7] Mojica J., Mo B., Ng A. (2017). Sphenopalatine Ganglion Block in the Management of Chronic Headaches. Curr. Pain Headache Rep..

[8] Cady R.K., Saper J.R., Dexter K., Cady R.J., Manley H.R. (2015). Long-Term Efficacy of a Double-Blind, Placebo-Controlled, Randomized Study for Repetitive Sphenopalatine Blockade With Bupivacaine vs Saline With the Tx 360 ® Device for Treatment of Chronic Migraine. Headache: J. Head Face Pain.

[9] Candido K.D., Massey S.T., Sauer R., Darabad R.R., Knezevic N.N. (2013). A novel revision to the classical transnasal topical sphenopalatine ganglion block for the treatment of headache and facial pain. Pain Physician.

[10] Dance L., Aria D., Schaefer C., Kaye R., Yonker M., Towbin R. (2017). Safety and efficacy of sphenopalatine ganglion blockade in children: Initial experience. J. Vasc. Interv. Radiol..

[11] Tian Medical [Homepage on the Internet]. https://europe.tianmedical.com/.

[12] Schaffer J.T., Hunter B.R., Ball K.M., Weaver C.S. (2015). Noninvasive Sphenopalatine Ganglion Block for Acute Headache in the Emergency Department: A Randomized Placebo-Controlled Trial. Ann. Emerg. Med..

[13] Hurst H., Bolton J. (2004). Assessing the clinical significance of change scores recorded on subjective outcome measures. J. Manip. Physiol. Ther..

[14] Loncarić-Katušin M., Milošević M., Žilić A., Mišković P., Majerić-Kogler V., Žunić J. (2016). Practical chronic pain assessment tools in clinical practice. Acta Clin. Croat..

[15] Martin S.R., Zeltzer L.K., Seidman L.C., E Allyn K., A Payne L. (2019). Caregiver–Child Discrepancies in Reports of Child Emotional Symptoms in Pediatric Chronic Pain. J. Pediatr. Psychol..

[16] Kashikar-Zuck S., Flowers S.R., Claar R.L., Guite J., Logan D.E., Lynch-Jordan A.M., Palermo T.M., Wilson A.C. (2011). Clinical utility and validity of the Functional Disability Inventory among a multicenter sample of youth with chronic pain. Pain.

[17] Raniti M.B., Waloszek J.M., Schwartz O., Allen N.B., Trinder J. (2018). Factor structure and psychometric properties of the Pittsburgh Sleep Quality Index in community-based adolescents. Sleep.

[18] Erwin A.M., Bashore L. (2017). Subjective Sleep Measures in Children: Self-Report. Front. Pediatr..

[19] Piagkou M., Demesticha T., Troupis T., Vlasis K., Skandalakis P., Makri A., Mazarakis A., Lappas D., Piagkos G., O Johnson E. (2011). The Pterygopalatine Ganglion and its Role in Various Pain Syndromes: From Anatomy to Clinical Practice. Pain Pract..

[20] Ho K.W.D., Przkora R., Kumar S. (2017). Sphenopalatine ganglion: Block, radiofrequency ablation and neurostimulation—A systematic review. J. Headache Pain.

[21] Kirkpatrick D.L., Townsend T., Walter C., Clark L., Alli A., Fahrbach T., Madarang E.J., Lemons S., Reeves A., Collins Z. (2020). Lidocaine Versus Bupivacaine in the Treatment of Headache with Intranasal Sphenopalatine Nerve Block. Pain Physician.

[22] Fares H.E., Mohamed S.A., Badawy F.A., Abdelfattah K.A.M. (2020). Comparative study between lidocaine 2%, lidocaine 5% and bupivacaine 0.5% in transnasal sphenopalatine ganglion block for the treatment of postdural puncture headache. Indian J. Appl. Res..

[23] Eccleston C., Fisher E., Cooper T.E., Grégoire M.-C., Heathcote L., Krane E., Lord S.M., Sethna N.F., Anderson A.-K., Anderson B. (2019). Pharmacological interventions for chronic pain in children: An overview of systematic reviews. Pain.

[24] Kacperski J., Kabbouche M.A., O’Brien H.L., Weberding J.L. (2016). The optimal management of headaches in children and adolescents. Ther. Adv. Neurol. Disord..

[25] Choinière M., Peng P., Gilron I., Buckley N., Williamson O., Janelle-Montcalm A., Baerg K., Boulanger A., Di Renna T., Finley G.A. (2020). Accessing care in multidisciplinary pain treatment facilities continues to be a challenge in Canada. Reg. Anesthesia Pain Med..

[26] Fisher E., Law E., Dudeney J., Palermo T.M., Stewart G., Eccleston C. (2018). Psychological therapies for the management of chronic and recurrent pain in children and adolescents. Cochrane Database Syst. Rev..

[27] Tang W.-X., Zhang L.-F., Ai Y.-Q., Li Z.-S. (2018). Efficacy of Internet-delivered cognitive-behavioral therapy for the management of chronic pain in children and adolescents: A systematic review and meta-analysis. Medicine.

[28] Powers S.W., Hershey A.D., Coffey C.S. (2017). The Childhood and Adolescent Migraine Prevention (CHAMP) Study: “What Do We Do Now?”. Headache J. Head Face Pain.

[29] Chan V.W., McCabe E.J., MacGregor D.L. (2009). Botox Treatment for Migraine and Chronic Daily Headache in Adolescents. J. Neurosci. Nurs..

[30] Hershey A.D., Powers S.W., Vockell A.-L.B., LeCates S., Kabbouche M., Maynard M.K. (2001). PedMIDAS: Development of a questionnaire to assess disability of migraines in children. Neurology.

